# Promotion of presynaptic filament assembly by the ensemble of *S. cerevisiae* Rad51 paralogues with Rad52

**DOI:** 10.1038/ncomms8834

**Published:** 2015-07-28

**Authors:** William A. Gaines, Stephen K. Godin, Faiz F. Kabbinavar, Timsi Rao, Andrew P. VanDemark, Patrick Sung, Kara A. Bernstein

**Affiliations:** 1 Department of Molecular Biochemistry and Biophysics, Yale University School of Medicine, New Haven, Conneticut 06510, USA; 2 Department of Microbiology and Molecular Genetics, University of Pittsburgh School of Medicine, 5117 Centre Avenue, UPCI Research Pavilion, G5.c, Pittsburgh, Pennsylvania 15217, USA; 3 Department of Biological Sciences, University of Pittsburgh, Pittsburgh, Pennsylvania 15260, USA

## Abstract

The conserved budding yeast Rad51 paralogues, including Rad55, Rad57, Csm2 and Psy3 are indispensable for homologous recombination (HR)-mediated chromosome damage repair. Rad55 and Rad57 are associated in a heterodimer, while Csm2 and Psy3 form the Shu complex with Shu1 and Shu2. Here we show that Rad55 bridges an interaction between Csm2 with Rad51 and Rad52 and, using a fully reconstituted system, demonstrate that the Shu complex synergizes with Rad55–Rad57 and Rad52 to promote nucleation of Rad51 on single-stranded DNA pre-occupied by replication protein A (RPA). The *csm2–F46A* allele is unable to interact with Rad55, ablating the ability of the Shu complex to enhance Rad51 presynaptic filament assembly *in vitro* and impairing HR *in vivo.* Our results reveal that Rad55–Rad57, the Shu complex and Rad52 act as a functional ensemble to promote Rad51-filament assembly, which has important implications for understanding the role of the human RAD51 paralogues in Fanconi anaemia and cancer predisposition.

## Abstract (web summary)

Homologous repair of DNA double strand breaks in *Saccharomyces cerevisiae* is dependent on several conserved Rad51 paralogs. Here the authors provide biochemical evidence that Rad55-Rad57 synergistically interacts with the Shu complex to promote Rad51 filament formation and homology directed repair.

## Graphical abstract

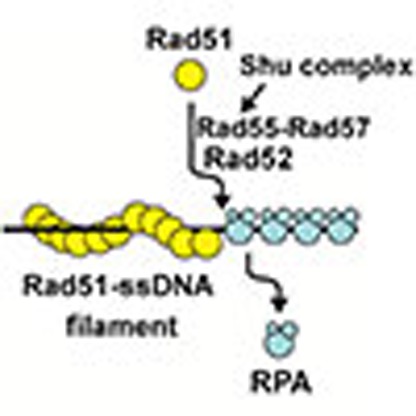

Homologous recombination (HR) represents a major tool for repairing injured replication forks, chromosomes with DNA double-strand breaks (DSBs), telomere lengthening and ensuring the accurate disjunction of homologous chromosomes in meiosis I (refs 1, 2). Defective HR underlies human diseases including cancer and Fanconi anaemia^3^,^4^,^5^,^6^,^7^. An important conserved HR step is the formation of a Rad51-single-stranded DNA (ssDNA) nucleoprotein filament, also called the presynaptic filament, at DNA damage. In yeast, Rad51 presynaptic filament assembly on ssDNA pre-occupied by RPA, the ubiquitous single-strand DNA-binding protein, is facilitated by Rad52 (or by BRCA2 in humans) and a heterodimeric complex of the Rad51 paralogues Rad55 and Rad57 (refs 2, 8). In conjunction with the DNA motor protein Rad54, the presynaptic filament mediates homology search and strand invasion, and the resultant DNA joint, the D-loop, is resolved by mechanistically distinct pathways to a crossover or non-crossover product.

Recent studies identified the yeast Shu complex as a novel factor that promotes Rad51-dependent repair by an undefined mechanism^9^,^10^,^11^,^12^,^13^. The Shu complex consists of four subunits that include two Rad51 paralogues (Csm2 and Psy3)—Shu1 and Shu2 (ref. 10) and is conserved in humans^9^,^14^,^15^. The Shu complex was originally identified during a screen for mutants that suppress the slow growth of *top3*Δ cells and it was shown to act in the *RAD52* epistasis group to promote HR^10^,^12^. The Shu complex similarly suppresses the HU and MMS sensitivity of *sgs1*Δ cells, placing it at an early step of HR^10^. Here we present the first biochemical evidence for a synergistic role of the Shu complex with Rad55–Rad57 and Rad52 to promote Rad51 presynaptic filament formation on RPA-coated ssDNA. We find by yeast-two-hybrid (Y2H) that Rad55–Rad57 bridges an interaction between the Shu complex and Rad52 and Rad51. *In vitro*, the Shu complex stimulates Rad51-filament formation in the presence of Rad55–Rad57 and Rad52. Disruption of the Rad55–Csm2 interaction by use of the *csm2–F46A* allele blocks this Rad51-filament stimulation *in vitro* and impairs HR *in vivo.* Together, our data support a model wherein Csm2 and Rad55 bridge interactions among the Rad55–Rad57 heterodimer, the Shu complex and Rad52 to promote Rad51-dependent homology-directed DNA repair.

## Results

### The Shu complex promotes Rad51 presynaptic filament assembly

**Figure 1:**
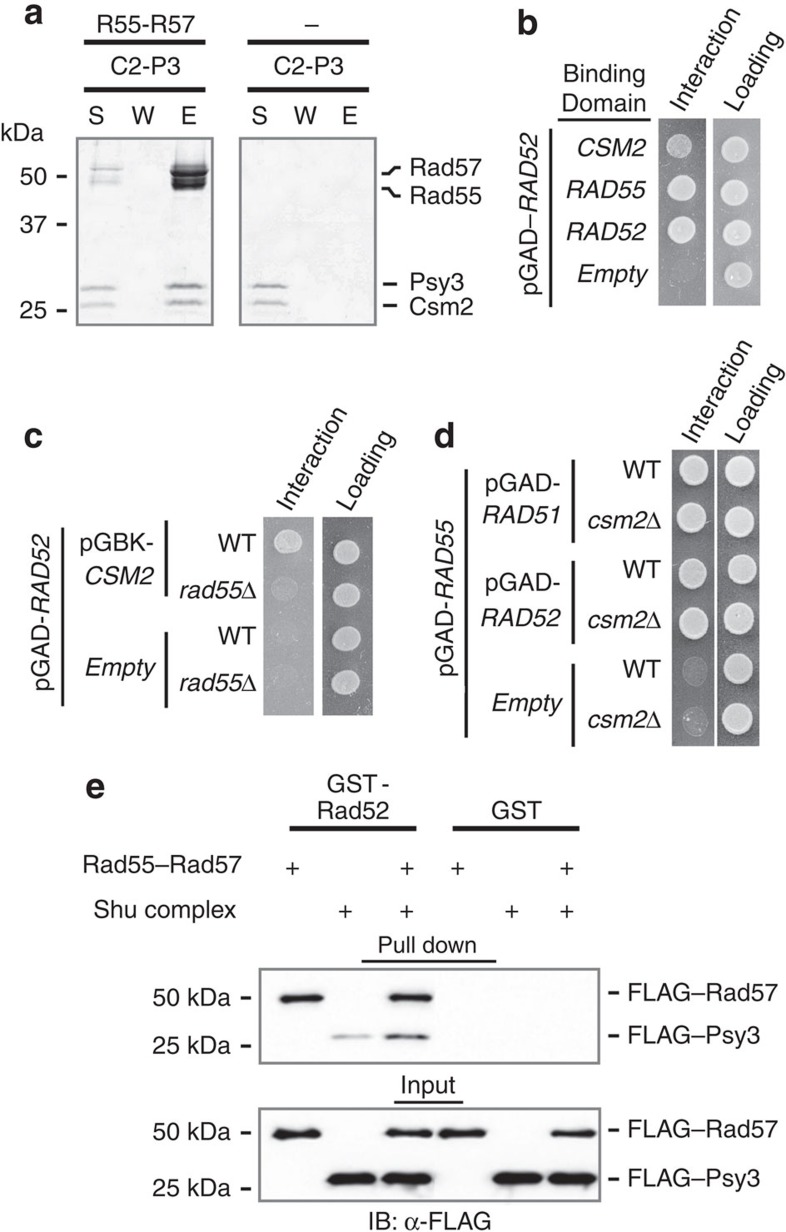
Interactions of Rad55–Rad57 with the Shu complex and Rad52. (**a**) Rad55–Rad57 was incubated with Csm2–Psy3 and the protein complex was captured through the (His)_6_ tag on Rad55 using Ni^2+^ resin. E, eluate from the resin; S, supernatant containing unbound proteins; W, wash of the resin. (**b**) Rad52 was tested for Y2H interaction with the indicated proteins. (**c**) The Y2H interaction between Rad52 and Csm2 was examined in wild-type or *rad55*Δ cells. (**d**) The Y2H interaction between Rad52 and Rad55 was examined in wild-type or *csm2*Δ cells. (**e**) GST-tagged Rad52 was mixed with Rad55–Rad57, Shu complex, or both, and protein complexes were captured on glutathione resin. Immunoblotting for the FLAG tag on Rad57 and Psy3 was used to identify Rad55–Rad57 and Shu complex retained on the resin.

We first focused on the purified Csm2–Psy3 heterodimer (Supplementary Fig. 1a), as the known protein interaction and DNA-binding activities occur through these subunits, including our previous findings that Csm2 associates with Rad55 in the Y2H system^16^. Indeed, we found by affinity pull-down that Csm2–Psy3 interacts with Rad55–Rad57 (Fig. 1a and Supplementary Fig. 2a). Similarly, the Shu complex (Csm2–Psy3–Shu1–Shu2) interacts with Rad55–Rad57 by pull-down (Supplementary Fig. 2a). We next asked whether Csm2 would interact with Rad52 by Y2H analysis (Fig. 1b). As shown before, association of Csm2 with Rad55 and self-interaction of Rad52 were seen^16^,^17^,^18^, and, importantly, Csm2 and Rad55 interacted with Rad52 (Fig. 1b). While the Csm2–Rad52 interaction was abolished in *rad55*Δ cells, the Rad52–Rad55 interaction remained without *CSM2* (Fig. 1c,d). We employed biochemical pull-down to validate these interactions and found that, consistent with the Y2H results above, while GST-tagged Rad52 could interact with Rad55–Rad57 (Fig. 1e), its interaction with the Shu complex was negligible. We attempted to validate that Rad55–Rad57 bridges Rad52 to Shu complex by performing the GST pull-down on a mixture of GST–Rad52, Rad55–Rad57 and Shu complex. However, the degree to which Shu complex is pulled down by Rad52 is either not affected or only modestly affected by the presence of Rad55–Rad57 (Fig. 1e), perhaps because the associations are too labile for the tripartite assembly to persist through washing steps. In agreement with prior Y2H results^16^,^17^, pull-down analysis revealed that Rad55–Rad57 interacts with Rad51 while Csm2–Psy3 does so only weakly (Supplementary Fig. 2b). Addition of Csm2–Psy3 does not attenuate Rad55–Rad57 interaction with Rad51, suggesting the interactions are non-competitive. In addition, we have tested RPA in pull-downs and found that it may interact weakly with Rad55–Rad57, though this association could not be validated by Y2H (Supplementary Fig. 2c).

It has been proposed that the Shu complex and Rad55–Rad57 act to promote Rad51 presynaptic filament formation by antagonizing the Srs2 helicase^19^, which negatively regulates HR by disrupting the presynaptic filament^20^,^21^. Cells lacking Srs2 therefore accumulate ssDNA-bound Rad51. Since Rad54 enhances the ability of the Rad51 presynaptic filament to catalyse DNA strand invasion^22^, *srs2*Δ *rad54*Δ double mutants are inviable, likely because they accumulate long-lived presynaptic filaments. Thus, *srs2*Δ *rad54*Δ inviability is suppressed by disruption of *RAD52,* which limits presynaptic filament formation^23^. Similarly, disruption of *RAD55* or *CSM2* suppresses *srs2*Δ *rad54*Δ inviability^24^. To expand on these findings, we asked whether disruption of *RAD55* or *CSM2* suppresses *srs2*Δ *rad54*Δ inviability to the same extent as *RAD52* deletion by tetrad dissection and spore analysis, and indeed it does (Supplementary Fig. 3, *P* value<0.05 by *χ*^2^-test). Thus, the Shu complex and Rad55–Rad57 do not act solely as negative regulators of Srs2 and can function independently of Rad54.

**Figure 2:**
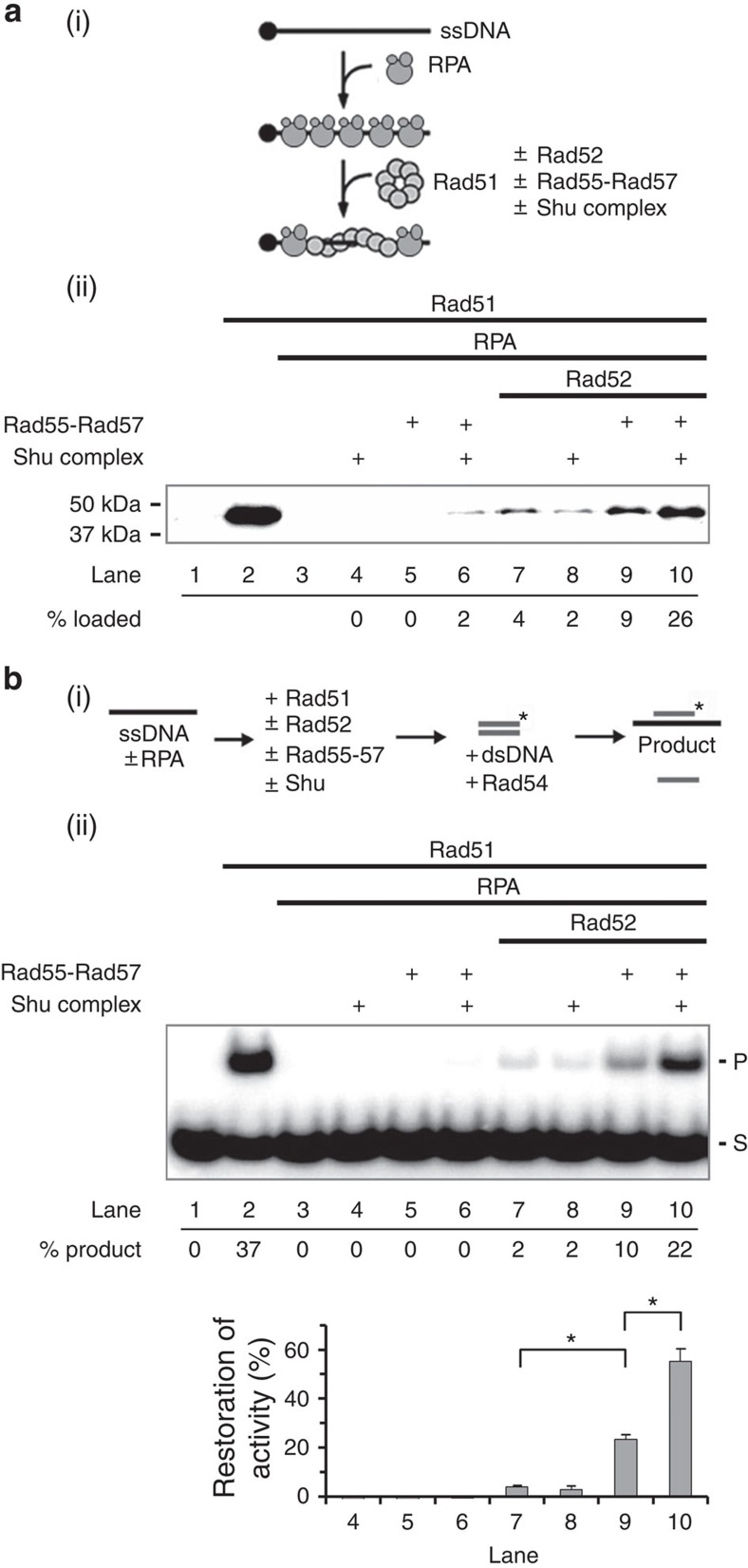
Enhancement of Rad51 loading onto RPA-coated ssDNA by the Shu complex and Rad55–Rad57. (**a**) (i) Schematic of the Rad51 loading assay. (ii) Magnetic bead resin with immobilized ssDNA was pre-incubated with RPA, then Rad51 was added along with combinations of Rad52, Shu complex and Rad55–Rad57. The amount of Rad51 retained on the ssDNA was determined by immunoblotting. (**b**) (i) Schematic of the DNA strand exchange assay. (ii) RPA-coated ssDNA was incubated with Rad51 and combinations of Rad52, Shu complex and Rad55–Rad57, mixed with radiolabelled homologous double-stranded DNA (dsDNA) and Rad54 and then analysed. P, DNA strand exchange product; S, dsDNA substrate. S.d. are plotted as error bars (*n*=3) and ‘*’ indicates significance.

We used several complementary approaches to assess the role of the Shu complex in Rad51 presynaptic filament assembly (Fig. 2a,b, Supplementary Fig. 4). Specifically, we monitored Rad51 loading onto RPA-coated ssDNA bound to magnetic beads (Fig. 2a) and also examined homologous DNA strand exchange activity (Fig. 2b). In the absence of RPA, Rad51 readily gained access to the bead-immobilized ssDNA and efficiently catalysed the exchange of homologous DNA strands (Fig. 2a,b, lane 2). However, RPA on ssDNA strongly attenuated Rad51 loading efficiency and DNA strand exchange (Fig. 2a,b, lane 3). Under the reaction conditions, the Shu complex and Rad55–Rad57 alone or together were insufficient to overcome the inhibitory effect of RPA (Fig. 2a,b, lanes 4–6). The addition of Rad52 permitted Rad51 loading onto RPA-coated ssDNA, but, because of the limiting conditions employed, the amount of ssDNA-associated Rad51 was only a fraction of that achieved when RPA was absent (Fig. 2a,b, lane 7 compared with 2). Rad51 loading in the presence of Rad52 was stimulated by the addition of Rad55–Rad57 but not the Shu complex (Fig. 2a,b, lanes 7 compared with 8 and 9). Importantly, adding Shu complex together with Rad52 and Rad55–Rad57 led to a marked stimulation of Rad51 loading and DNA strand exchange activity (Fig. 2a,b, lane 10). The enhancement of Rad51 loading by Rad55–Rad57 and the Shu complex is most prominent at both 18 and 23 °C and less pronounced at 30 °C (Fig. 2b and Supplementary Fig. 5a,b). This is consistent with *rad55*Δ cells exhibiting a more severe DNA damage sensitivity at lower temperatures^19^,^25^. Since we found that Shu complex stimulates Rad51-filament assembly, we next visualized the nucleoprotein products of Rad51 loading using electron microscopy (EM). Consistent with our biochemical data, we observed a significantly greater number of Rad51 filaments in reactions where Rad55–Rad57 and Shu complex were added together with Rad52 (Supplementary Fig. 4). Collectively, these results reveal a synergistic action of Rad52 and the two Rad51 paralogue complexes in enabling utilization of RPA-coated ssDNA for presynaptic filament assembly.

We found that the Csm2–Psy3 heterodimer is just as adept as the full Shu complex in the enhancement of presynaptic filament assembly in the presence of Rad55–Rad57, suggesting that Shu1 and Shu2, which are not Rad51 paralogues, may not be directly involved in presynaptic filament assembly (Supplementary Fig. 6a). The DNA strand exchange reactions performed in Fig. 2b contained Rad54 to accelerate the rate of product formation^26^, but similar results were obtained without Rad54 (Supplementary Fig. 6b). Rad55–Rad57 and the Shu complex do not appear to directly enhance the activities of Rad51 or Rad54 since DNA strand exchange reactions lacking RPA are not stimulated by addition of Rad55–Rad57 and Shu complex (Supplementary Fig. 6c).

**Figure 3:**
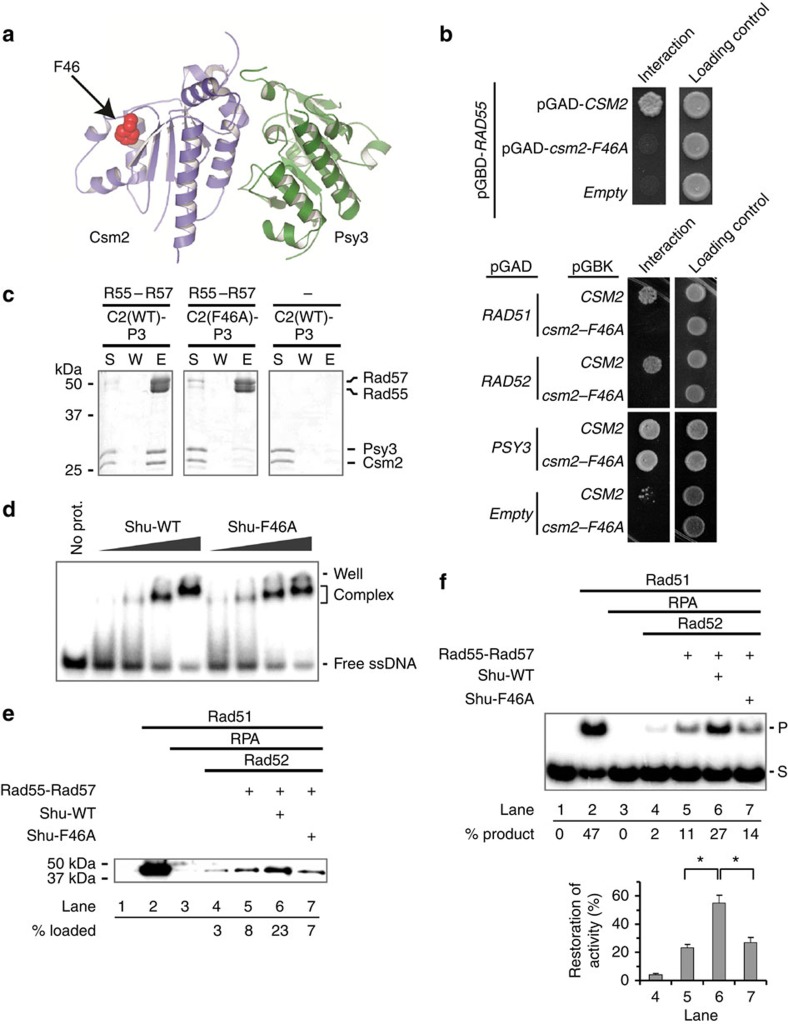
Impairment of physical interaction and functional synergy of Shu complex with Rad55–Rad57 by the *csm2–F46A* mutation. (**a**) Cartoon view of the Csm2–Psy3 heterodimer^29^, with Csm2 in blue and Psy3 in green. Csm2–F46 is highlighted in red. (**b**) Y2H analysis for interaction of Csm2 and csm2–F46A with Rad55, Rad51, Rad52 and Psy3. (**c**) Pull-down assay (as in Fig. 1a) to examine interaction of (His)_6_-Rad55–Rad57 with Csm2–Psy3 harbouring csm2–F46A. (**d**) Analysis of the DNA-binding activity of Shu complex harbouring csm2–F46A. (**e**) Rad51 loading and (**f**) DNA strand exchange assays were carried out as in Fig. 2 to evaluate the efficacy of Shu complex harbouring csm2–F46A in the promotion of presynaptic filament assembly. S.d. are plotted as error bars (*n*=3) and ‘*’ indicates significance.

By X-ray crystallography, several groups have determined the structure of Csm2–Psy3 (refs 27, 28, 29). We searched for solvent exposed residues that could participate in interaction with a partner molecule such as Rad55. We applied surface triplet propensity analysis^30^ to this structure^29^ to reveal a potential protein interaction surface on Csm2 (Supplementary Fig. 7a), which is highly conserved in numerous fungal homologues (Supplementary Fig. 7b). The phenylalanine 46 to alanine (F46A) (Fig. 3a and Supplementary Fig. 7) mutation was made, as changing this surface residue is unlikely to affect the stability or folding of Csm2. Importantly, we found that the F46A mutation disrupts the Y2H interaction of Csm2 with Rad55 but not Psy3 (Fig. 3b).

We purified Csm2–Psy3 containing the csm2–F46A mutant and verified that it is impaired for Rad55–Rad57 interaction (Fig. 3c) but is proficient in DNA binding (Shu-F46A; Fig. 3d) and complex formation with Shu1–Shu2 (Supplementary Fig. 1). Consistent with Rad55 bridging the interactions between Csm2 with Rad51 and Rad52, we found that the mutant Csm2 does not interact with Rad51 or Rad52 (Fig. 3b), and, importantly, that Shu-F46A is unable to enhance Rad51 presynaptic filament assembly in the bead-based Rad51 loading assay or DNA strand exchange reaction (Fig. 3e,f, compare lane 6 with 7). These results provide the first evidence that the physical interaction between Csm2 and Rad55–Rad57 is functionally significant and are congruent with our finding that the Shu complex synergizes with Rad55–Rad57 in Rad51 presynaptic filament assembly.

**Figure 4:**
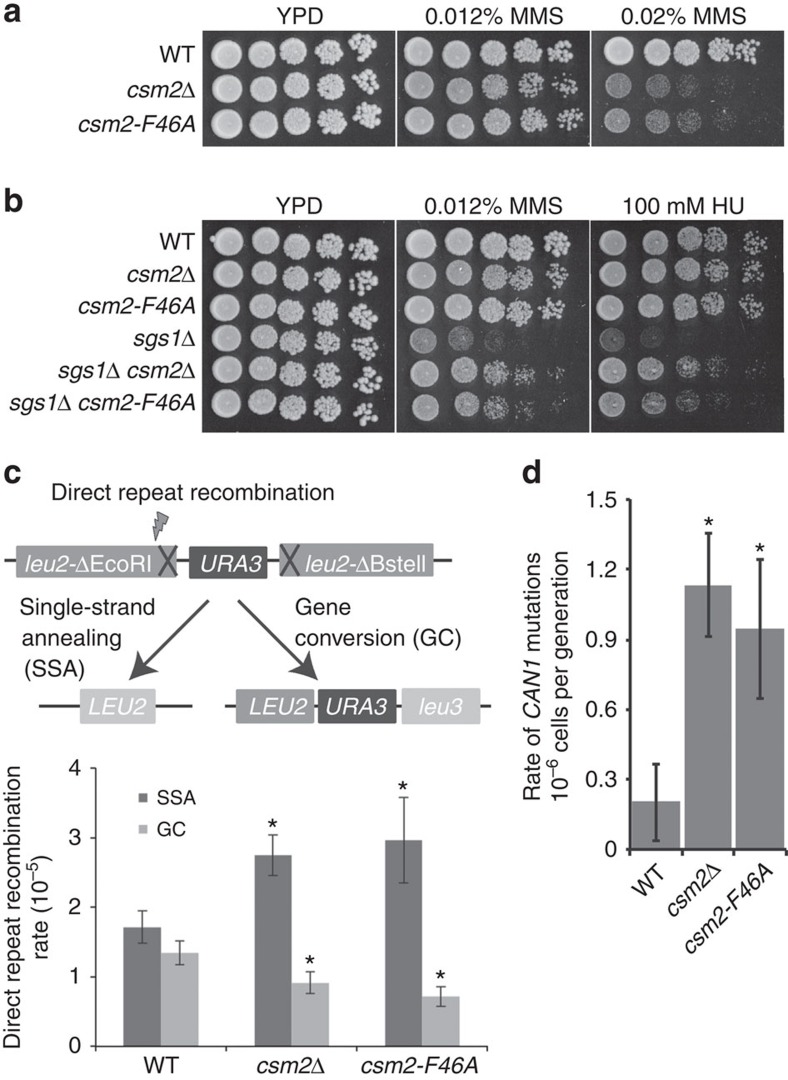
Impairment of homologous recombination by the *csm2–F46A* mutation. (**a**) *csm2–F46A* cells are as sensitive to MMS as a *csm2*Δ mutant. Cultures were fivefold serially diluted onto YPD medium with the indicated dose of MMS and incubated for 2 days at 30 °C. (**b**) Similar to *csm2*Δ, *csm2–F46A* alleviates the MMS and HU sensitivity of an *sgs1*Δ mutant. Cells of the indicated genotypes were fivefold serially diluted and tested for sensitivity to 0.012% MMS or 100 mM HU. (**c**) WT, *csm2*Δ or *csm2-F64A* cells harbouring a direct repeat HR reporter (*leu2*-ΔEcoRI*::URA3::leu2*-ΔBstEII) were tested for spontaneous rates of Rad51-dependent gene conversion (GC) and Rad51-independent single-strand annealing (SSA) as described^16^. The rates of GC are significantly decreased in both *csm2–F46A* and *csm2*Δ strains (*P*<0.01 by student’s *t*-test) with a corresponding increase in SSA relative to WT strains (*P*<0.02 by student’s *t*-test). S.d. are plotted as the error bars (*n*=3) and ‘*’ indicates significance. (**d**) Like *csm2*Δ cells, *csm2–F46A* cells exhibit an elevated mutation rate in a canavanine mutagenesis assay. S.d. are plotted as error bars (*n*=5) and ‘*’ indicates significance.

To determine if the Csm2–Rad55 interaction is important for HR, we integrated *csm2–F46A* into the endogenous *CSM2* locus and found that the mutant cells are almost as sensitive to MMS as *csm2*Δ cells (Fig. 4a). Furthermore, since previous studies demonstrated that the Shu complex acts upstream of Sgs1, we tested if the *csm2–F46A* mutation suppresses the MMS and HU sensitivity of an *sgs1*Δ mutant like *csm2*Δ and found that it does (Fig. 4b). We next employed a direct repeat recombination assay^16^,^31^ to reveal that *csm2–F46A* cells are as impaired as the *csm2*Δ mutant in Rad51-mediated gene conversion (Fig. 4c), with a corresponding increase in Rad51-independent single-strand annealing events (Fig. 4c^16^; *P* value<0.02 by student’s *t*-test). Consistent with disruption of other HR factors^32^, using a canavanine mutagenesis assay we found that *csm2–F46A* cells accumulate spontaneous mutations like *csm2*Δ (refs 10, 13) (Fig. 4d). Together, our results demonstrate that the interaction between Csm2 and Rad55 is indispensable for the biological efficacy of the Shu complex.

## Discussion

Regulation of Rad51 presynaptic filament formation is essential for HR-mediated repair of damaged DNA and stalled replication forks. Mutations in human mediators of Rad51 presynaptic filament assembly, which is, the hRAD51 paralogues (RAD51B, RAD51C, RAD51D, XRCC2, XRCC3) or BRCA2, can result in a predisposition to numerous cancers^3^,^4^,^5^,^6^,^7^. Whether the Rad51 mediators function independently or in a cooperative manner has remained unknown^33^,^34^,^35^. In budding yeast, Rad52 and the Rad55–Rad57 complex have been shown biochemically to promote presynaptic filament formation in isolation. We have furnished evidence that while the Shu complex is devoid of recombination mediator activity, it synergizes with Rad55–Rad57 and Rad52 to mediate the assembly of Rad51 on RPA-coated ssDNA (Supplementary Fig. 8). Our work supports a model in which Rad55–Rad57 bridges an interaction between Rad52 and the Shu complex (Fig. 1) that is functionally important for Rad51 presynaptic filament assembly (Fig. 2, Supplementary Figs 4 and 8). Our study represents the first *in vitro* reconstitution of Rad51 presynaptic filament assembly being mediated by an ensemble of seven proteins (Rad52, Rad55–Rad57 and Shu1–Shu2-Csm2–Psy3). Furthermore, we have revealed the importance of the Csm2–Rad55 interaction in promoting HR in cells (Fig. 4) and in the facilitation of Rad51 presynaptic filament assembly and homologous DNA pairing and strand exchange *in vitro* (Fig. 3). Genetic evidence suggests that the Shu complex acts primarily in the repair of stalled replication forks^13^, and while Shu1 and Shu2 are required for efficient fork repair *in vivo* they are dispensable for Rad51 loading *in vitro* (Supplementary Fig. 6a). One possibility is that the Shu complex interaction with Rad55–Rad57 enables its association with replication fork-derived DNA intermediates and subsequently Shu1 and Shu2 would be necessary for DNA repair progression. Although the function of Shu1–Shu2 remains elusive, previous work suggested that they may regulate Shu complex binding to complex DNA structures produced during HR^29^, or Shu complex interactions with other HR regulators, such as Srs2 (refs 8, 19, 36). Intriguingly, the arrangement of the human BCDX2 RAD51 paralogue complex is similar to the Shu complex with Rad55–Rad57 (Supplementary Fig. 9). Given the conservation of the HR machinery, mechanistic insights into the yeast Rad51 paralogues will provide a model for understanding how the human paralogues promote HR. For example, the human RAD51 paralogues may work with BRCA2, the functional equivalent of yeast Rad52, in presynaptic filament assembly^37^. This postulated function of the human RAD51 paralogues is likely important for cancer avoidance^3^,^4^,^5^,^6^,^7^.

## Methods

### Yeast culture methods

The strains used are listed in Supplementary Table 1. Yeast crosses, transformations, tetrad dissections and media preparation were carried out using standard methods^16^. Briefly, yeast diploids were acquired by streaking two haploid yeast strains together on a yeast peptone dextrose (YPD) plate and replica plating onto a double-selective medium that would allow selection for diploids. For tetrad dissections, diploid yeast were plated onto sporulation medium and allowed to sporulate for 72 h prior to dissection on a Zeiss AX10 dissection scope. Tetrads where grown for 2 days before being genotyped by replica plating onto corresponding selective medium. For plasmid transformations, 50 ml of yeast were grown to mid-log phase (0.3–0.6 OD_600_) in YPD medium, pelleted, resuspended in 100 mM lithium-acetate with 1 × Tris-EDTA solution with 300 ng of plasmid, and then incubated at 30 °C for 30 min with 34% PEG. Cells were heat-shocked at 42 °C for 15 min and then plated onto selective medium for each plasmid. The *csm2–F46A* mutation was introduced into integration, Y2H and expression vectors by site-directed mutagenesis using primers Csm2.F46A. Forward (5′-GATGCCACAAGCTCAGCTCCGCTAAGTCAATTCC-3′) and Csm2.F46A. Reverse (5′-GGAATTGACTTAGCGGAGCTGAGCTTGTGGCATC-3′). The integration vector was made using primers Csm2.Integration.HindIII (5′-CCCAAGCTTAACAATTCCTCTCTGAGTTGAAA-3′) and Csm2.Integration.MfeI (5′-CCCCAATTGTATCCTTTAAATAAAACCTGTTTTTCCC-3′) to amplify *CSM2* with promoter and terminator from genomic DNA. The amplified DNA was cut with HindIII and MfeI and ligated into yiPLAC211 cut with HindIII and EcoRI.

### Y2H analysis

For Y2H analysis, haploid yeast containing either the AD or BD plasmids were mated overnight on YPD, then selected on double-selective media as described above to acquire diploids with both Y2H plasmids (SC-LEU-TRP). For Y2H experiments, cells were grown overnight in double-selective liquid medium (SC-LEU-TRP) and then 5 μl of yeast at a concentration of 0.5 OD_600_ were plated on selective medium (SC-LEU-TRP-HIS or SC-LEU-TRP)^16^ and incubated at 30 °C for 2 days.

### Serial dilutions

Fivefold serial dilutions were performed with a starting 0.5 OD_600_ using standard techniques^16^. Briefly, yeast were grown overnight, diluted to 0.2 OD_600_ and incubated at 30 °C for 3 h prior to serial dilution.

### Mitotic recombination assays

Mitotic recombination assays were performed^16^,^31^, and recombination rates and standard deviations were calculated using Lea and Coulson’s method of the mean^38^. Briefly, for each genotype, nine independent colonies were inoculated into synthetic complete (SC) medium and grown to saturation overnight at 30 °C. Cells were plated onto SC-LEU medium to examine recombination events and SC medium assess cell viability and cell number. After 2 days of growth, the colonies on the SC-LEU plates were replica-plated onto SC–URA medium and SC+5-FOA medium to score for Rad51-dependent and -independent recombination events, respectively. Each experiment was performed in quadruplicate with s.d. plotted and significance determined by *t*-test.

### Canavanine mutagenesis assay

For each trial, individual *CAN1* colonies from WT, *csm2*Δ and *csm2–F46A* cells were grown in 5 ml YPD medium overnight at 30 °C, diluted 1:10 and 250 μl were plated onto SC-ARG+CAN plates. To determine the number of cells plated, the culture was further diluted 1:10,000 and 120 μl were plated onto YPD plates. The plates were incubated at 30 °C for 2 days before colonies were counted. The relative rate of *CAN1* inactivation for each strain is reported as an average of five experiments with s.d. plotted.

### Suppression of *rad54*Δ *srs2*Δ synthetic lethality

To analyse the suppression of *rad54*Δ *srs2*Δ synthetic lethality, a diploid yeast strain heterozygous for the indicated genes was sporulated for 72 h and tetrad dissected onto rich medium and incubated for 2 days at 30 °C. Three hundred and sixty-one tetrads were genotyped and assessed for viability. The *χ*^2^-analysis was used to determine significance for which genotypes were associated with inviability.

### Protein expression and purification

Rad51, Rad52, RPA and Rad54 proteins were prepared as follows^39^,^40^,^41^:

The Rad55–Rad57 complex was expressed in *Saccharomyces cerevisiae* strain JEL1 (ref. 42) by using the pESC-URA vector (Agilent) harbouring (His)_6_-tagged *RAD55* and FLAG-tagged *RAD57* genes; the affinity tags are fused to the N-termini of Rad55 and Rad57 proteins. Cells were grown at 30 °C until OD_660_ of 0.7, then induced for protein expression by the addition of galactose to 2%, cultured for an additional 18 h and harvested by centrifugation. All the subsequent steps were carried out at 4 °C in Buffer T (25 mM Tris-HCl, pH 7.4, 10% glycerol, 0.5 mM EDTA, 0.01% IGEPAL CA-630 (Sigma), 1 mM DTT) was used throughout protein purification. Importantly, the inclusion of ATP and Mg^2+^ during lysis (with 5 mM ATP and 2 mM Mg^2+^) and purification steps (with 2 mM of each) helps minimize the aggregation of Rad55–Rad57 and precipitation. To prepare cell lysate, a 40 g cell pellet was resuspended in 160 ml of buffer with 500 mM KCl, 10% sucrose, 0.1% IGEPAL and protease inhibitors (1 mM phenylmethylsulfonyl fluoride and 5 μg ml^−1^ each of aprotinin, chymostatin, leupeptin and pepstatin) then lysed by grinding with dry ice followed by sonication. After ultracentrifugation (100,000*g* for 1 h), the clarified lysate was incubated with 2 ml of Ni^2+^-NTA affinity resin (Qiagen) for 30 min with gentle mixing. The resin was poured into a column and washed using buffer with 500 mM KCl and 20 mM imidazole, followed by elution using wash buffer supplemented with 250 mM imidazole. The eluate was then mixed with 0.5 ml anti-FLAG affinity resin (Sigma) for 2 h with gentle mixing. The resin was poured into a column and washed using buffer with 200 mM KCl, followed by elution using wash buffer supplemented with 0.3 mg ml^−1^ FLAG peptide. The protein pool was diluted with an equal volume of buffer T and fractionated in a 1 ml Mono Q column with a 30 ml gradient of 100–700 mM KCl, collecting 0.5 ml fractions. The peak fractions of Rad55–Rad57 (eluting at ∼290 mM KCl) were pooled, concentrated in an Amicon Ultra micro-concentrator (Millipore) to 0.6 ml, and fractionated in a 24 ml SuperDex 200 size exclusion column (GE Healthcare) in buffer with 300 mM KCl, collecting 0.5 ml fractions. The peak fractions of Rad55–Rad57 (eluting at appropriate position for monodisperse, heterodimeric complex) were pooled, concentrated in an Amicon Ultra micro-concentrator to 150 μl, snap-frozen in liquid nitrogen and stored at −80 °C. The yield of highly purified Rad55–Rad57 was ∼150 μg.

The Shu complex was expressed in *Escherichia coli* (Rosetta [DE3]) by co-transforming cells with the pET-Duet vector harbouring (His)_6_-tagged Shu1 and MBP-tagged Shu2 and the pRSF-Duet vector harbouring Strep-tagged Csm2 and FLAG-tagged Psy3. All the affinity tags are fused to the N terminus of proteins. Cells were grown in 2 × LB broth supplemented with 0.1 mM ZnCl_2_ at 37 °C until OD_600_ of 0.8, and protein expression was induced by the addition of IPTG (isopropyl β-D-1-thiogalactopyranoside) to 0.2 mM and shifted to growth at 16 °C for 16 h. Cells were harvested by centrifugation. All the subsequent steps were conducted at 4 °C in buffer T. For lysate preparation, a 40 g cell pellet was resuspended in 200 ml of buffer with 300 mM KCl and protease inhibitors as above and then lysed by sonication. After ultracentrifugation (100,000*g* for 1 h), the clarified lysate was incubated with 2 ml of Ni^2+^-NTA affinity resin (Qiagen) for 30 min with gentle mixing. The resin was poured into a column and washed using buffer with 150 mM KCl and 10 mM imidazole, followed by elution using wash buffer supplemented with 200 mM imidazole. The eluate was then mixed with 2 ml amylose affinity resin (NEB) for 2 h with gentle mixing. The resin was poured into a column and washed with buffer with 150 mM KCl. Tobacco etch virus protease (10 μg) was mixed with the resin to cleave at the linker between MBP and Shu2 and thus permit release of the Shu complex from the amylose resin; this incubation was performed overnight at 4 °C. The released Shu complex was mixed with 0.7 ml anti-FLAG affinity resin for 2 h with gentle mixing. The resin was poured into a column and washed using buffer with 150 mM KCl, followed by elution using wash buffer supplemented with 0.3 mg ml^−1^ FLAG peptide. The eluate was concentrated in an Amicon Ultra micro-concentrator to 0.6 ml and fractionated in a 24 ml SuperDex 200 column in buffer with 150 mM KCl, collecting 0.5 ml fractions. For both WT and F46A forms, Shu complex eluted at the position for monodisperse tetrameric protein complex. The peak fractions were pooled, concentrated in an Amicon Ultra micro-concentrator to 100 μl, snap-frozen in liquid nitrogen and stored at −80 °C. The yield of highly purified Shu complex was 65 μg.

### Affinity pull-down assays

Csm2–Psy3 (0.5 μg) was incubated with or without His-tagged Rad55–Rad57 (0.5 μg) in 30 μl of buffer C (20 mM MOPS, pH 7.2, 150 mM KCl, 10% glycerol, 0.01% IGEPAL, 1 mM DTT, 1 mM ATP, 2 mM MgCl_2_, 15 mM imidazole) for 60 min at 4 °C. The reactions were mixed with 4 μl of Ni^2+^-NTA resin for 30 min at 4 °C. After washing the resin five times with 150 μl of buffer C with 200 mM KCl, bound proteins were eluted with 2% SDS. The supernatant, elution, and wash fractions were analysed by 15% SDS–PAGE followed by Coomassie Blue staining. For pull-down via GST–Rad52, proteins were captured on glutathione resin using the same procedure as above but using buffer with 100 mM KCl and no imidazole, then the various fractions were immunoblotted with α-FLAG antibody (Sigma Aldrich, catalogue #A8592; 1:3,000 dilution) to detect the FLAG-tagged Shu complex and Rad55–Rad57. The input samples shown represent one-thirtieth of the total input. Blot images of the full gel exposure for this experiment are shown in Supplementary Fig. 10a.

### Rad51 loading onto ssDNA immobilized on magnetic beads

RPA (0.4 μM) was added to biotinylated dT 83-mer ssDNA (1.4 μM nucleotides) coupled to magnetic streptavidin beads (Roche) in 10 μl of buffer A (35 mM MOPS, pH 7.2, 50 mM KCl, 1 mM DTT, 2 mM ATP, 5 mM MgCl_2_, 100 μg ml^−1^ BSA) at 37 °C for 10 min. Excess RPA was removed by magnetic separation. Then, a mixture consisting of the indicated combination of Rad51, Rad52, Rad55–Rad57 and Shu complex was added to the RPA-coated ssDNA resin in 10 μl buffer A. The protein concentrations used were 0.75 μM Rad51, 0.06 μM Rad52, 0.11 μM Rad55–Rad57 and 0.23 μM Shu complex. The reactions were incubated at 18 °C (except where stated otherwise) for 90 min with periodic agitation. The beads were briefly washed twice with 10 μl buffer A. Proteins were eluted with 2% SDS and Rad51 amount was determined by immunoblotting with α-Rad51 antibody^43^ (1:3,000 dilution). Full blot images for this experiment are shown in Supplementary Fig. 10b.

### Homologous DNA pairing and strand exchange reaction

Oligonucleotide-based DNA pairing and strand exchange assay was conducted as described previously^44^ with slight modifications. The 80-mer ssDNA oligonucleotide (1.6 μM nucleotides) was incubated with RPA (0.13 μM) in buffer A at 37 °C for 10 min. Then, a mixture consisting of the indicated combination of Rad51, Rad52, Rad55–Rad57 and Shu complex was incorporated. The protein concentrations used were 0.64 μM Rad51, 0.05 μM Rad52, 0.14 μM Rad55–Rad57 and 0.14 μM Shu complex. The reactions were incubated at 18 °C (except where stated otherwise) for 90 min, then spermidine (4 mM final concentration) and ^32^P-labeled homologous 40-mer double-stranded DNA (0.8 μM base pairs final concentration) with Rad54 (0.035 μM final concentration) and an ATP regenerating system (20 mM creatine phosphate, 30 μg ml^−1^ creatine kinase) were added to complete the reaction (final volume of 12.5 μl). The reactions were incubated for 30 min at 18 °C, deproteinized by treatment with 1% SDS and 1 mg ml^−1^ proteinase K for 5 min at 37 °C, and then subjected to electrophoresis in a 10% polyacrylamide gel in TAE buffer (40 mM Tris acetate, pH 7.4, 0.5 mM EDTA). Radiolabelled DNA species were visualized and quantified by phosphorimaging. Please note: a ‘no ATP’ control could not be performed due to the presence of ATP in the protein storage buffer for Rad55–Rad57.

### Electron microscopy

The reactions were set-up similarly to the DNA strand exchange assay for examining Rad51 loading efficiency, except that a longer ssDNA substrate (150-mer) was used and BSA was not included in buffer A. After a 1.5 h incubation at 18 °C, the reaction was applied to a glow-discharged, carbon-coated EM grid (Ted Pella, Inc.) and stained with 2% uranyl acetate. The EM images were acquired on an FEI Tecnai T12 transmission electron microscope at magnification of 30,000 with 20 images sampled per grid. About 200 to 400 nucleoprotein particles were counted for each reaction condition.

### DNA-binding assay

The Shu complex (0.05, 0.10, 0.20 and 0.40 μM) was incubated with radiolabelled 83-mer ssDNA (1 μM nucleotides) in buffer B (20 mM MOPS, pH 7.2, 1 mM DTT, 150 mM KCl, 5 mM MgCl_2_ and 2.5 mM ATP) for 10 min at 30 °C. DNA species were resolved by electrophoresis in an 8% polyacrylamide gel run in TA buffer (40 mM Tris acetate, pH 7.4) and then visualized and quantified by phosphorimaging.

## Additional information

**How to cite this article:** Gaines, W. A. *et al.* Promotion of presynaptic filament assembly by the ensemble of *S. cerevisiae* Rad51 paralogues with Rad52. *Nat. Commun.* 6:7834 doi: 10.1038/ncomms8834 (2015).

## Supplementary Material

Supplementary Information Supplementary Figures 1-10 and Supplementary Table 1
