# Supplementary material for: Promotion of presynaptic filament assembly by the ensemble of S. cerevisiae Rad51 paralogues with Rad52
Source: Nat Commun. 2015 Jul 28;6:7834. doi: 10.1038/ncomms8834 (PMC4525180; doi:10.1038/ncomms8834)
Supplement: Supplementary Information — Supplementary Figures 1-10 and Supplementary Table 1 [file ncomms8834-s1.pdf]

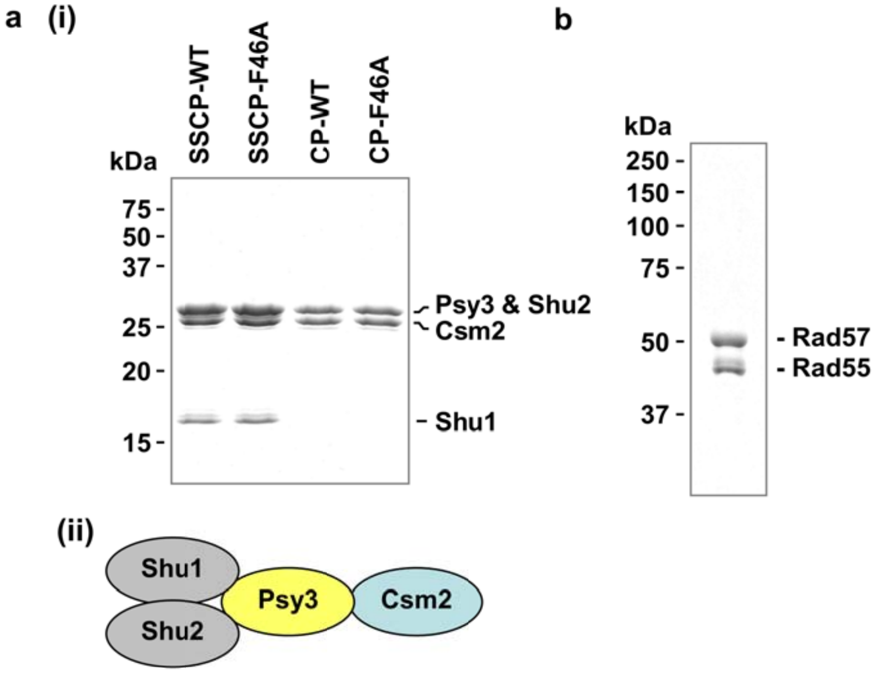


**Supplementary Figure 1. Purity analysis of Shu complex and Rad55-Rad57.**

**a**, (i) SDS-PAGE analysis of purified Shu (SSCP; Shu1-Shu2-Csm2-Psy3) complex and Csm2-Psy3 (CP; Csm2-Psy3) complex with either wild-type Csm2 or the csm2-F46A mutant. **a**, (ii) Schematic depiction of the arrangement of subunits within the Shu complex. **b**, SDS-PAGE analysis of purified Rad55-Rad57 complex.

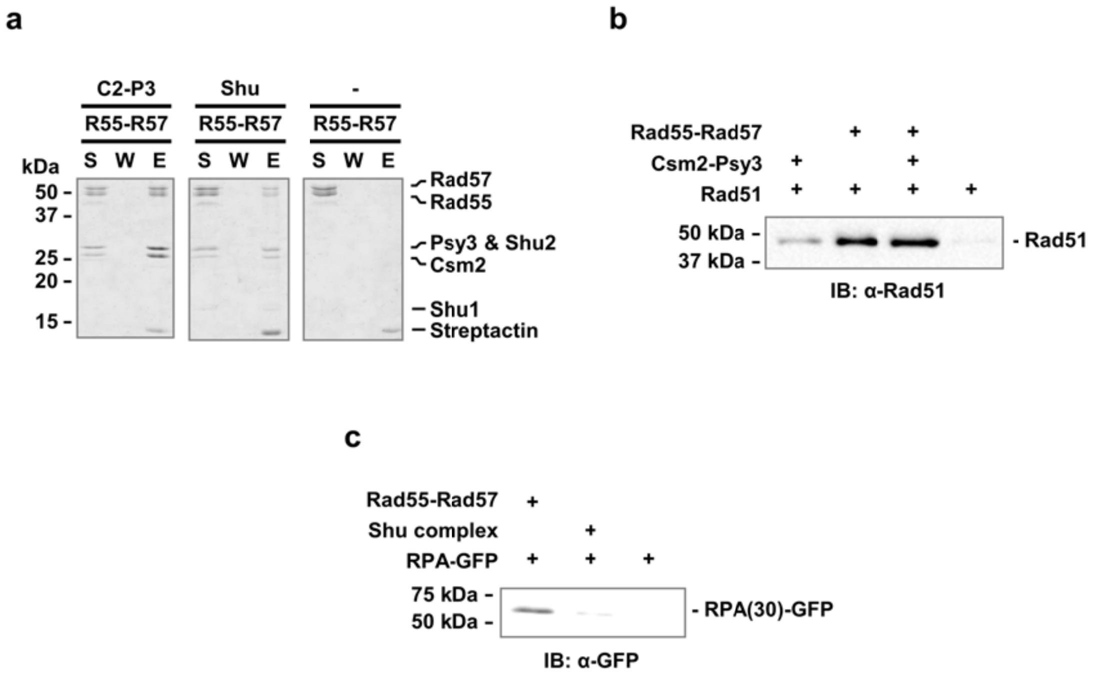


**Supplementary Figure 2. Interactions of Rad55-Rad57 with the Shu complex, Rad51, and RPA.**

**a**, Physical interaction of Csm2-Psy3 or Shu complex with Rad55-Rad57 was revealed by pull-down in which proteins were captured on Streptactin resin via Csm2-Strep. Analysis followed the procedure described in Figure 1a. **b**, Interactions of Rad51 with Rad55-Rad57 or Csm2-Psy3 was assessed by pull-down. Complexes were captured on anti-FLAG resin via the FLAG tag on Psy3 or Rad57. The elution fractions were

immunoblotted for Rad51. **c**, Interactions of RPA with Rad55-Rad57 or Shu complex was assessed by pull-down. Complexes were captured on anti-FLAG resin via the FLAG tag on Psy3 or Rad57. The elution fractions were immunoblotted for GFP fused to the 30 kDa subunit of RPA. GFP tagged RPA was used to facilitate immunodection of the weak interaction.

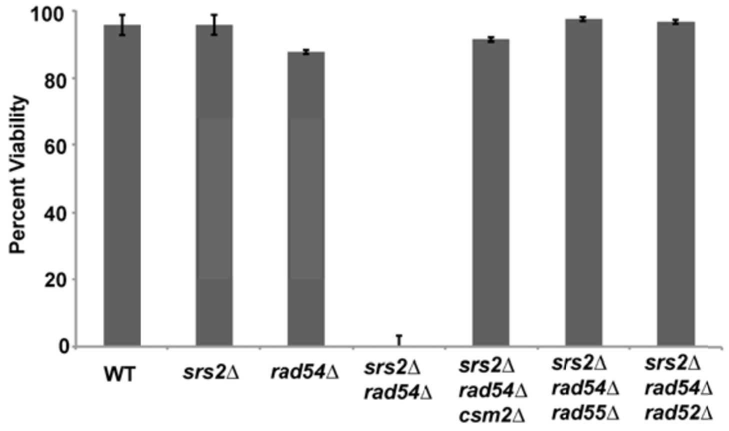


**Supplementary Figure 3. Genetic analyses of *csm2*Δ for functions independent of *RAD54* and *SRS2.***

Deletion of *CSM2, RAD55*, or *RAD52* suppresses the synthetic lethality of *srs2*Δ *rad54*Δ. Viability is plotted with standard error for 361 distinct tetrads shown as error-bars.

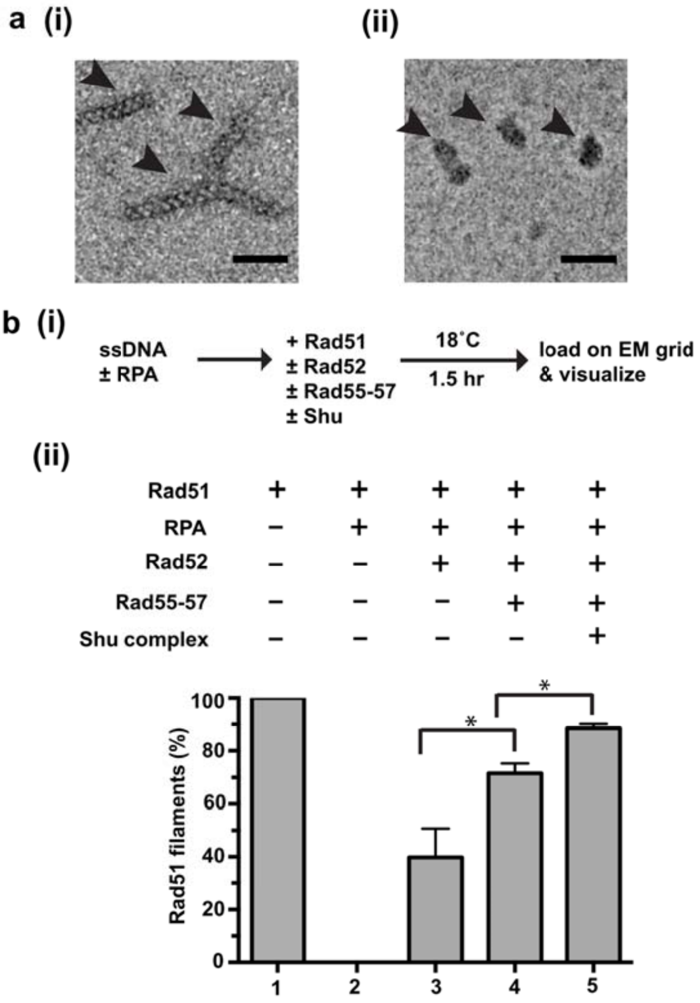


**Supplementary Figure 4. Electron microscopic visualization of the nucleoprotein complexes formed via Shu complex and Rad55-Rad57 facilitated loading of Rad51.** **a**, Representative images of Rad51 (i) and RPA (ii) nucleoprotein complexes with ssDNA as visualized by negatively stained EM (scale bars: 50 nm) **b**, (i) Schematic of the Rad51 loading assay employed for EM visualization. **b**, (ii) RPA-coated ssDNA was incubated with Rad51 and combinations of Rad52, Shu complex, and Rad55-Rad57, then analyzed by EM. The graphed values represent the portion of all nucleoprotein

complexes observed (both Rad51 and RPA) that were identified as Rad51 filaments under each condition. Standard deviations are plotted as error bars (n=3) and (*) indicates significance.

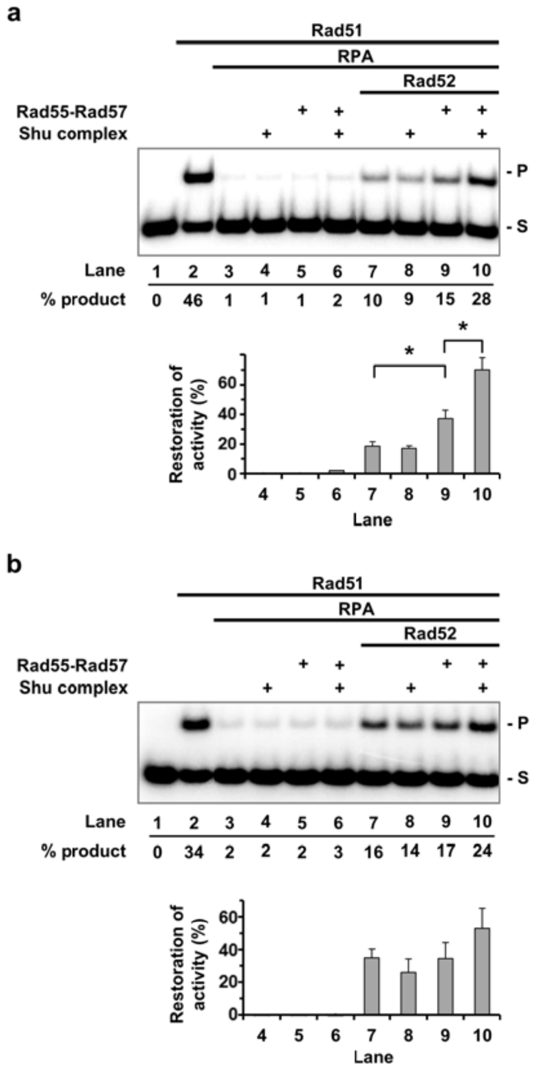


**Supplementary Figure 5. Analysis of the effect of Rad55-Rad57 and Shu complex on Rad51 loading at 23°C and 30°C.**

DNA strand exchange assay was carried out (as in Fig. 2b) to assess the effect of Rad55-Rad57 and Shu complex on Rad51 loading at reaction temperatures of **a**, 23°C and **b,** 30°C.

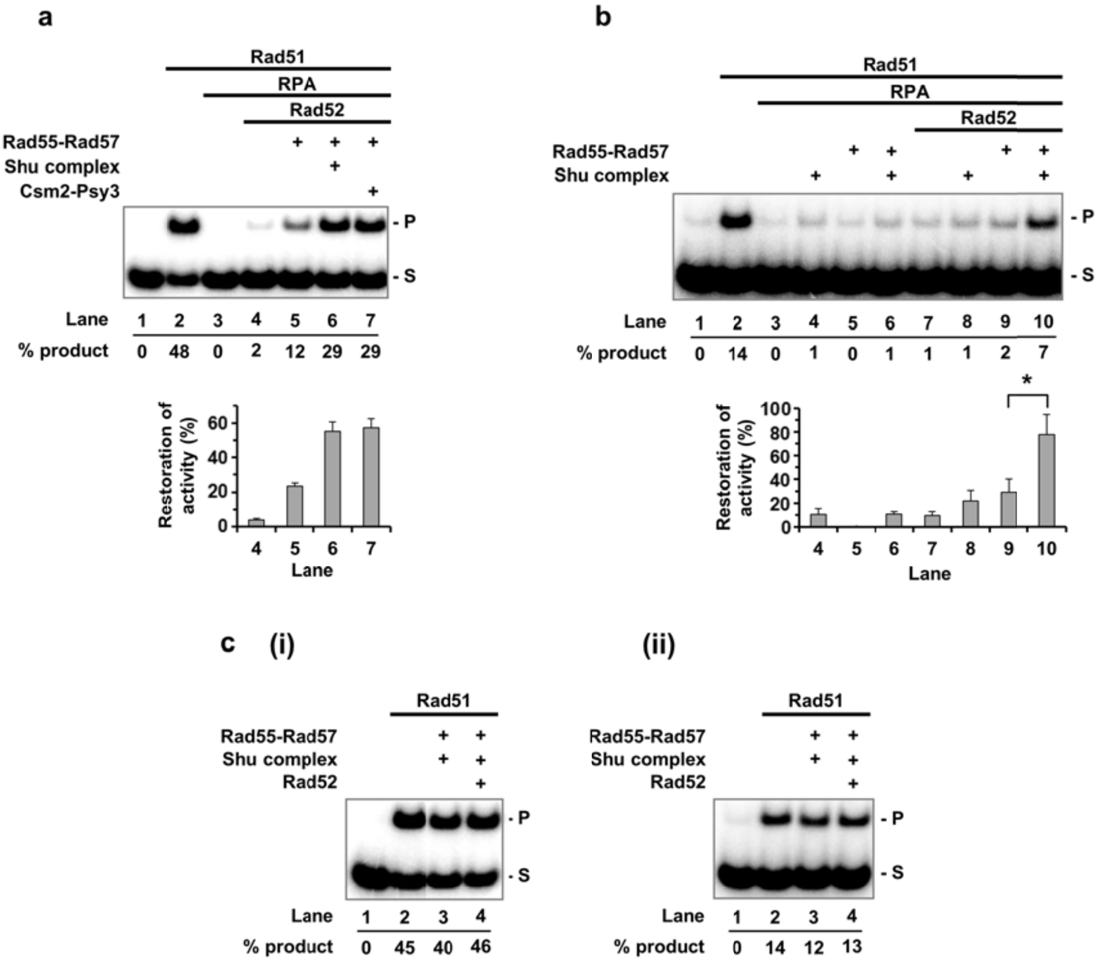


**Supplementary Figure 6. Analysis of Shu complex and Csm2-Psy3 complex in Rad51-mediated DNA strand exchange.**

**a**, Results showing that Csm2-Psy3 is just as adept as Shu complex in enabling Rad51 to utilize an RPA-coated ssDNA substrate for DNA strand exchange. Reactions were

carried out as in Figure 2b (i.e. at 18°C with Rad54 present). **b**, Results showing that omission of Rad54 does not affect the ability of Rad55-Rad57 and Shu complex to promote DNA strand exchange with an RPA-coated ssDNA substrate. The reactions were carried out as in Figure 2b, except that Rad54 was omitted and the reaction time upon dsDNA addition extended to 10 hrs. **c**, Results showing that Rad55-Rad57 and Shu complex do not stimulate DNA strand exchange activity of Rad51 when free ssDNA is used as substrate. DNA strand exchange reactions were performed without RPA present. In (i), Rad54 was added to the reactions and a reaction time of 30 min was used, similarly to Figure 2b. In (ii), Rad54 was omitted and a reaction time of 10 hrs was used, as in panel **b**.

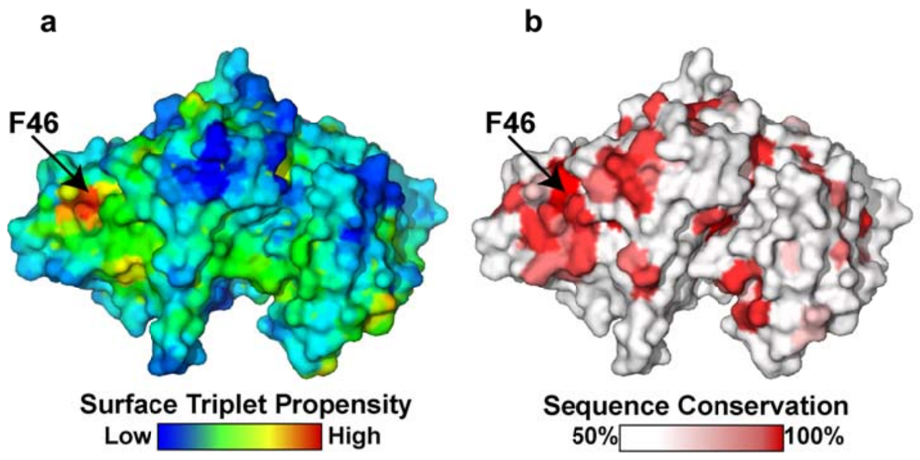


**Supplementary Figure 7. Analysis of the Csm2-Psy3 structure to identify potential protein-protein interaction surfaces.**

**a**, Surface Triplet Propensity (STP) mapped onto the surface of the Csm2-Psy3 heterodimer. STP values, which evaluate the local chemical environment of an atom to predict its likelihood of participating in a protein-protein interface[30] were calculated for all atoms in the Csm2-Psy3 heterodimer and colored as a heat map with residues having a higher predicted score in red. The location of Csm2 F46 is indicated. **b**, Sequence conservation from an alignment of 21 fungal orthologs was mapped onto the surface of

the Csm2-Psy3 heterodimer. Invariant residues (including Csm2 F46) are shown in red while positions with less than 50% identity are colored white.

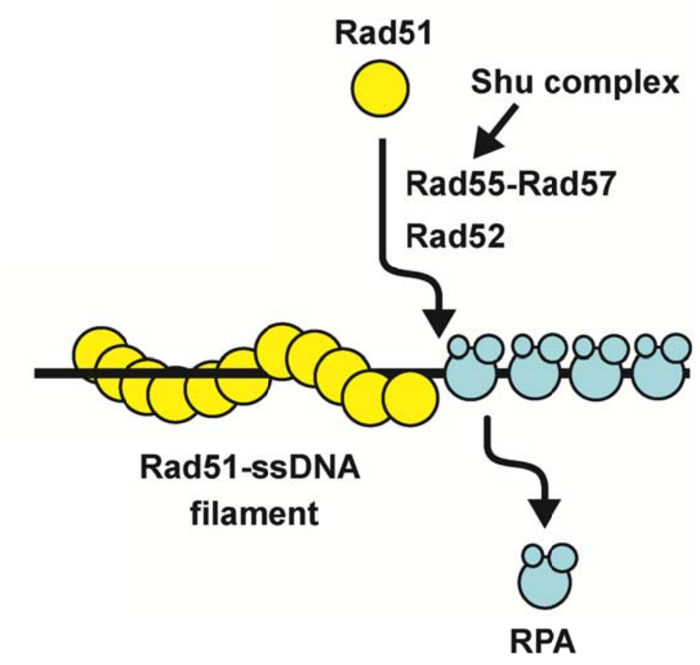


**Supplementary Figure 8. Model for Shu complex function in presynaptic filament assembly.**

Rad52 and Rad55-Rad57 together promote the nucleation of Rad51 onto RPA-coated DNA to seed the assembly of the Rad51 presynaptic filament. Our results have revealed that Shu complex, via its interaction with Rad55-Rad57, enhances the efficiency of the filament assembly process.

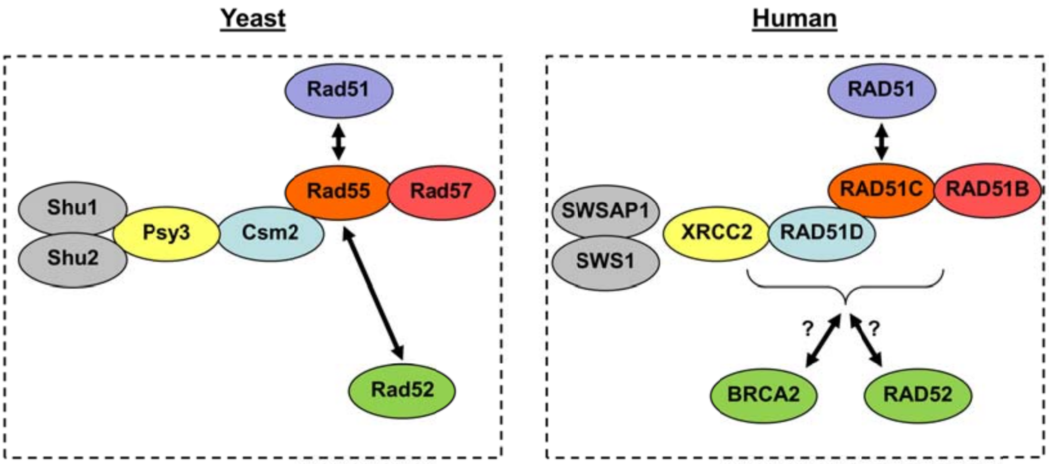


**Supplementary Figure 9. Comparison of human and yeast Rad51 paralog complexes.**

The RAD51 paralogs form the BCDX2 complex consisting of the RAD51B-RAD51C heterodimer associated with the RAD51D-XRCC2 heterodimer. Likewise, we have found that the yeast Rad51 paralogs associate into a similar higher order structure. Both human and yeast Rad51 paralogs interact with and functionally regulate Rad51. Given that the yeast Rad51 paralogs function with Rad52, it seems likely that the human RAD51

paralogs function in a similar manner with human RAD52 and/or BRCA2 in presynaptic filament assembly.

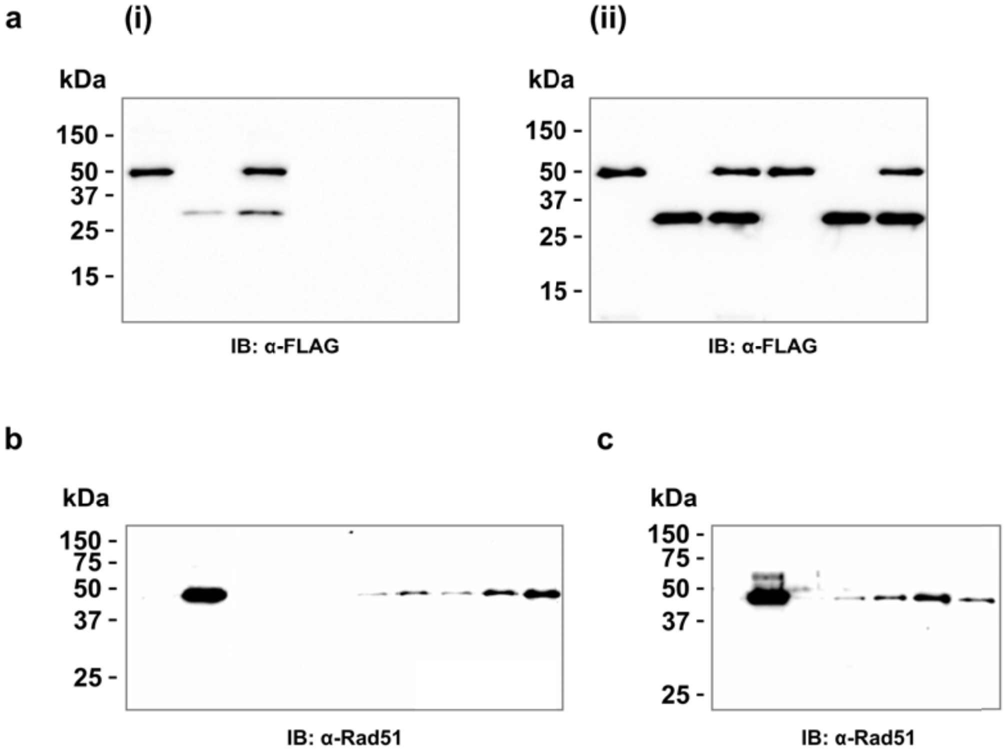


**Supplementary Figure 10. Uncropped images of immunoblots.**

The full blot images are displayed for the immunoblots presented in: **a**, Figure 1e for the pull-down (i) and input (ii) fractions; **b**, Figure 2a; **c**, Figure 3e.

**SUPPLEMENTARY TABLE 1: Strains and plasmids**

| Name | Description |
|---|---|
| PJ69-4A | *MAT***a** *trp1-901 leu2-3,112 ura3-52 his3-200 gal4Δ gal80Δ GAL2-ADE2 LYS2::GAL1-HIS3 met2::GAL7-lacZ* |
| PJ69-4*α* | *MAT* **α** *trp1-901 leu2-3,112 ura3-52 his3-200 gal4Δ gal80Δ GAL2-ADE2 LYS2::GAL1-HIS3 met2::GAL7-lacZ* |
| KBY212 | *MAT***a** *trp1-901 leu2-3,112 ura3-52 his3-200 gal4Δ gal80Δ GAL2-ADE2 LYS2::GAL1-HIS3 met2::GAL7-lacZ rad55::NatNT2* |
| KBY221 | *MAT* **α** *trp1-901 leu2-3,112 ura3-52 his3-200 gal4Δ gal80Δ GAL2-ADE2 LYS2::GAL1-HIS3 met2::GAL7-lacZ rad55::HphNT1* |
| KBY395 | *MAT* **α** *trp1-901 leu2-3,112 ura3-52 his3-200 gal4Δ gal80Δ GAL2-ADE2 LYS2::GAL1-HIS3 met2::GAL7-lacZ csm2::HphNT1* |
| W9100-2D | *MAT* **α** *ADE2 leu2-3,112 his3-11,15 ura3-1 TRP1 lys2Δ RAD5* |
| KBY619-7C | *MAT* **α** *csm2::KanMX4 LYS2 trp1-1* |
| KBY804-17D | *MAT***a** *LYS2 srs2::HIS3 csm2::KanMX4 rad55::NatNT2 rad52::URA3* |
| KBY363-3C | *MAT α* trp1-1 LYS2 rad54::LEU2 |
| KBY107-2D | *MAT***a** *csm2::KanMX LYS2* |
| KBY551 | *MAT α csm2-F46A* |
| KBY51-3B | *MAT***a** *sgs1::HIS3 trp1-1 LYS2* |
| KBY707-2A | *MAT***a** *sgs1::HIS3 bar1::LEU2 LYS2 csm2::KanMX4* |
| KBY611-1B | *MAT α sgs1::HIS3 LYS2 csm2-F46A* |
| KBY225-5B | *MAT***a** *leu2ΔEcoRI::URA3-HO::leu2ΔBsteII LYS2* |

| | |
|---|---|
| | *trp1-1* |
| KBY225-8D | *MAT α leu2ΔEcoRI::URA3-HO::leu2ΔBsteII csm2::kanMX LYS2 trp1-1* |
| KBY650-5C | *MAT***a** *csm2-F46A trp1-1 LYS2 leu2ΔEcoRI::URA3-HO::leu2ΔBstEII* |
| KBY233-6D | *MAT***a** *trp1-1 CAN1* |
| KBY614-3D | *MAT***a** *LYS2 trp1-1 csm2::KanMX4 CAN1* |
| KBY613-1B | *MAT***a** *trp1-1 LYS2 CAN1 Csm2-F46A* |
| ZOO347 | *pGAD-RAD52 (LEU, $AMP^R$)* |
| pWJ1481 | *pGBK-CSM2 (TRP, $AMP^R$)* |
| pGBD-RAD55 | *pGBD-RAD55 (TRP, $AMP^R$)* |
| pGBD-RAD52 | *pGBD-RAD52 (TRP, $AMP^R$)* |
| pGBD-C1 | *pGBD-C1(TRP, $AMP^R$)* |
| pGAD-RAD51 | *pGAD-RAD51 (LEU, $AMP^R$)* |
| MH34 | *ADH-RAD52 (TRP, $AMP^R$)* |
| pRS424 | *pRS424 (TRP, $AMP^R$)* |
| pWJ1476 | *pGAD-CSM2 (LEU, $AMP^R$)* |
| pKB212 | *pGAD-csm22-F46A (LEU, $AMP^R$)* |
| pKB44 | *pGBK-csm2-F46A (TRP, $KAN^R$)* |
| pGAD-C2 | *pGAD-C2 (LEU, $AMP^R$)* |
| pKB139 | yiPLAC211-Csm2-F46A |
| pET-DUET-S1S2 | Co-expression of Shu1 and Shu2 ($AMP^R$) |
| pRSF-DUET-C2P3 | Co-expression of Csm2 and Psy3 ($KAN^R$) |
| pESC-R55R57 | Co-expression of Rad55 and Rad57 (URA) |

All yeast strains are W303 background derivatives and *RAD5* (23) W1588 (24) except for PJ69-4A and PJ69-4 *α* (25). The KBY212, KBY221, and KBY395 strains were constructed in PJ69-4 *α* and PJ69-4A backgrounds, respectively. The strains are listed in the order they appear in the figures and text.
